# The Arachidonic Acid Metabolome Serves as a Conserved Regulator of Cholesterol Metabolism

**DOI:** 10.1016/j.cmet.2014.09.004

**Published:** 2014-11-04

**Authors:** Egon Demetz, Andrea Schroll, Kristina Auer, Christiane Heim, Josef R. Patsch, Philipp Eller, Markus Theurl, Igor Theurl, Milan Theurl, Markus Seifert, Daniela Lener, Ursula Stanzl, David Haschka, Malte Asshoff, Stefanie Dichtl, Manfred Nairz, Eva Huber, Martin Stadlinger, Alexander R. Moschen, Xiaorong Li, Petra Pallweber, Hubert Scharnagl, Tatjana Stojakovic, Winfried März, Marcus E. Kleber, Katia Garlaschelli, Patrizia Uboldi, Alberico L. Catapano, Frans Stellaard, Mats Rudling, Keiji Kuba, Yumiko Imai, Makoto Arita, John D. Schuetz, Peter P. Pramstaller, Uwe J.F. Tietge, Michael Trauner, Giuseppe D. Norata, Thierry Claudel, Andrew A. Hicks, Guenter Weiss, Ivan Tancevski

**Affiliations:** 1 Department of Internal Medicine VI, Innsbruck Medical University, Anichstrasse 35, 6020 Innsbruck, Austria; 2 Department of Internal Medicine, Angiology, Medical University of Graz, Auenbruggerplatz 15, 8036 Graz, Austria; 3 Department of Internal Medicine III, Innsbruck Medical University, Anichstrasse 35, 6020 Innsbruck, Austria; 4 Department of Ophthalmology and Optometry, Innsbruck Medical University, Anichstrasse 35, 6020 Innsbruck, Austria; 5 Department of Internal Medicine I, Innsbruck Medical University, Anichstrasse 35, 6020 Innsbruck, Austria; 6 Department of Pharmacology, Capital Medical University, Number 10 Xitoutiao, You An Men, 100069 Beijing, China; 7 Department of Pediatrics II, Innsbruck Medical University, Anichstrasse 35, 6020 Innsbruck, Austria; 8 Clinical Institute of Medical and Chemical Laboratory Diagnostics, Medical University of Graz, Auenbruggerplatz 15, 8036 Graz, Austria; 9 Department of Internal Medicine, Medical Clinic V, Mannheim Medical Faculty, University of Heidelberg, Theodor-Kutzer-Ufer 1–3, 68167 Mannheim, Germany; 10 Synlab Academy, Harrlachweg 1, 68163 Mannheim, Germany; 11 Center for the Study of Atherosclerosis, Bassini Hospital, via Gorki 50, 20092 Cinisello Balsamo Milan, Italy; 12 Department of Pharmacological and Biomolecular Sciences, Università Degli Studi di Milano, via Balzaretti 9, 20133 Milan, Italy; 13 IRCCS Multimedica, via Milanese 300, 20099 Sesto San Giovanni Milan, Italy; 14 Department of Pediatrics, University Medical Center Groningen, University of Groningen, Hanzeplein 1, 9700 RB Groningen, the Netherlands; 15 Department of Medicine and Department of Biosciences and Nutrition, Karolinska Institute at Karolinska University Hospital Huddinge, 14186 Stockholm, Sweden; 16 Department of Biological Informatics and Experimental Therapeutics, Graduate School of Medicine, Akita University, 1-1 Tegata Gakuen-machi, 010-8502 Akita City, Japan; 17 Department of Health Chemistry, University of Tokyo, 7-3-1 Hongo, Bunkyo, 113-8654 Tokyo, Japan; 18 Department of Pharmaceutical Sciences, St. Jude Children’s Research Hospital, 262 Danny Thomas Place, MS313, Memphis, TN 38105, USA; 19 Center for Biomedicine, European Academy Bozen/Bolzano (EURAC), Drususallee 1, 39100 Bolzano, Italy–Affiliated Institute of the University of Luebeck, Ratzeburger Allee 160, 23562 Luebeck, Germany; 20 Hans Popper Laboratory of Molecular Hepatology, Division of Gastroenterology and Hepatology, Department of Internal Medicine III, Medical University of Vienna, Waehringer Guertel 18-20, 1090 Vienna, Austria; 21 The Blizard Institute, Centre for Diabetes, Barts and The London School of Medicine & Dentistry, Queen Mary University, 4 Newark Street, E1 2AT London, UK

## Abstract

Cholesterol metabolism is closely interrelated with cardiovascular disease in humans. Dietary supplementation with omega-6 polyunsaturated fatty acids including arachidonic acid (AA) was shown to favorably affect plasma LDL-C and HDL-C. However, the underlying mechanisms are poorly understood. By combining data from a GWAS screening in >100,000 individuals of European ancestry, mediator lipidomics, and functional validation studies in mice, we identify the AA metabolome as an important regulator of cholesterol homeostasis. Pharmacological modulation of AA metabolism by aspirin induced hepatic generation of leukotrienes (LTs) and lipoxins (LXs), thereby increasing hepatic expression of the bile salt export pump Abcb11. Induction of Abcb11 translated in enhanced reverse cholesterol transport, one key function of HDL. Further characterization of the bioactive AA-derivatives identified LX mimetics to lower plasma LDL-C. Our results define the AA metabolome as conserved regulator of cholesterol metabolism, and identify AA derivatives as promising therapeutics to treat cardiovascular disease in humans.

## Highlights

• GWAS identifies *ALOX5* to associate with plasma cholesterol and HDL-C in humans
• Aspirin promotes reverse cholesterol transport (RCT) via Abcb11
• Lipoxins and leukotrienes regulate expression of Abcb11
• Lipoxin mimetics increase hepatic LDLr thereby lowering LDL-C

## Graphical Abstract

**Figure:**
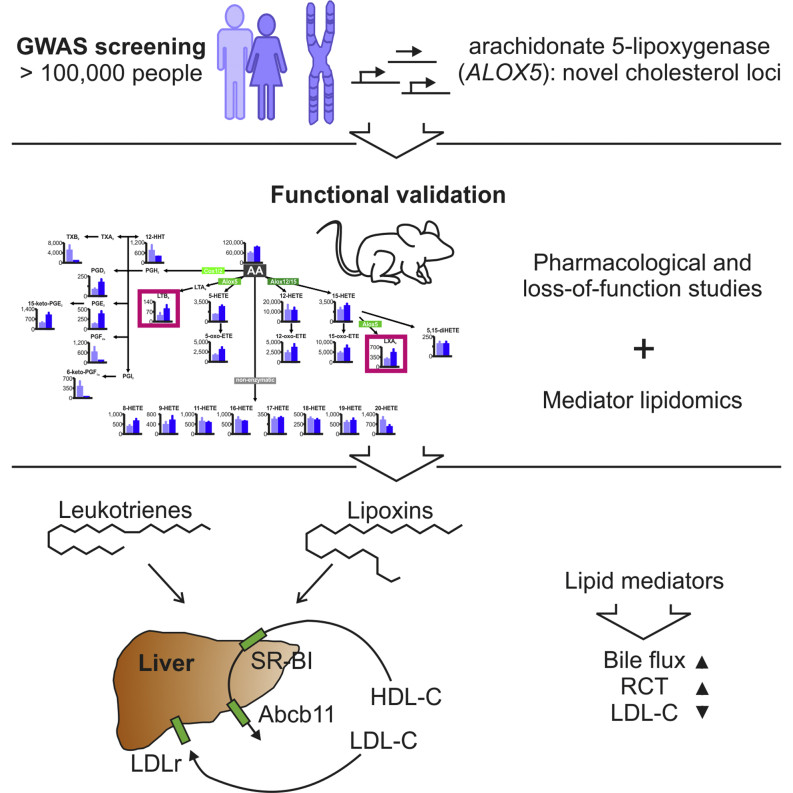

## Teaser

Omega-6 polyunsaturated fatty acids, including arachidonic acid (AA), have beneficial cardiovascular effects. Demetz et al. show that Alox5, a key enzyme of the AA pathway, regulates cholesterol in humans. Modulation of the AA pathways genetically or pharmacologically, with aspirin or bioactive AA-mimetics influences cholesterol metabolism including reverse cholesterol transport.

## Introduction

Atherosclerosis is still the leading cause of death in industrialized countries, and novel therapies to lower low-density lipoprotein cholesterol (LDL-C) are urgently needed. Additionally, any approach promoting the transport of excess cholesterol from plaque macrophages back to the liver via plasma high-density lipoprotein (HDL) for biliary and final fecal excretion is expected to prevent atherosclerosis, a mechanistic concept called reverse cholesterol transport (RCT) (Cuchel and Rader, 2006, Degoma and Rader, 2011, Rader and Daugherty, 2008). It is well known that dietary supplementation with omega-6 polyunsaturated fatty acids (omega-6 PUFAs) including arachidonic acid (AA) reduces the risk of cardiovascular disease (CAD) (Harris et al., 2009, Katan, 2009), which is in part attributable to the observation that increased AA plasma levels are associated with beneficial changes in LDL-C and HDL-C.

In humans, AA is metabolized into many potent bioactive compounds, such as (1) prostaglandins (PGs) and thromboxanes (TXs), (2) leukotrienes (LTs), and (3) lipoxins (LXs). Whereas PGs and TXs are formed by cyclooxygenases I and II (COX I/II), LTs are generated through the action of arachidonate 5-lipoxygenase (ALOX5), and LXs—an acronym of lipoxygenase interaction product—by the sequential cell-cell interaction of different lipoxygenases (McMahon and Godson, 2004, Serhan, 2007): LTA_4_, the intermediate of LT synthesis, is produced in neutrophils via ALOX5 and can be taken up by platelets and converted into LXs via ALOX12. 15S-Hydroxyeicosatetraenoic acid (15S-HETE) is synthesized in epithelial cells and monocytes via ALOX15, which can be further converted into LXs in leukocytes by ALOX5. Generation of LXs occurs also when 15-HETE accumulates in cell membranes of neutrophils, where it is converted into LXs (McMahon and Godson, 2004, Serhan, 2007). An additional route of LX biosynthesis emerges in cells exposed to aspirin. Aspirin acetylates COX II, changing its activity to a lipoxygenase. This generates 15R-HETE, which is finally converted into 15-epi-lipoxins via ALOX5 (McMahon and Godson, 2004, Serhan, 2007).

To date, the relative pathophysiological roles of lipoxygenases, LTs, and LXs have been extensively studied in inflammation where LTB_4_ exerts proinflammatory actions by promoting the recruitment of leukocytes to the site of insult. This is followed by an increase in anti-inflammatory eicosanoids LXA_4_ and its regioisomer LXB_4_, which mediate resolution of inflammation (Serhan, 2007). One important example of sustained chronic inflammation and failure of its resolution is found in atherosclerosis. It was proposed that any intervention leading to an increase in proresolving LXs may represent a novel therapeutic approach to interrupt the vicious circle of inflammation taking place in the arterial wall (Spite and Serhan, 2010). Aspirin constitutes such a pharmacological approach. Aspirin is a widely used drug for primary and secondary prevention of myocardial infarction, stroke, and unstable angina. By transforming the enzymatic properties of COX II into that of a lipoxygenase, aspirin was shown to increase the generation of LXs not only in different animal models of chronic inflammation, but also in humans, thereby inhibiting the accumulation of leukocytes at sites of inflammation (Spite and Serhan, 2010).

Intriguingly, evidence from genome-wide association studies (GWASs) revealed a robust association between single nucleotide polymorphisms (SNPs) of *ALOX5* and of *5-lipoxygenase activating protein* (*FLAP*) with the risk of myocardial infarction, obesity, and stroke (Helgadottir et al., 2004, Mehrabian et al., 2005, Peters-Golden and Henderson, 2007). Confirmation from independent studies is, however, warranted to fully support the genetic association between ALOX5 and CAD.

So far, neither the relative role of different AA-metabolizing enzymes, including lipoxygenases, nor the potential impact of their main metabolites, i.e., LTB_4_ and LXs, on cholesterol metabolism has been systematically investigated. Here, we elucidate the relative roles of LTs and LXs on cholesterol homeostasis by combining data from GWAS analysis in humans, mediator lipidomics, and loss-of-function studies in mice. We identify LX mimetics as promising therapeutics to lower plasma LDL-C and to treat atherosclerosis.

## Results

### Identification of ALOX5 as Regulator of HDL-C in Humans

**Figure 1:**
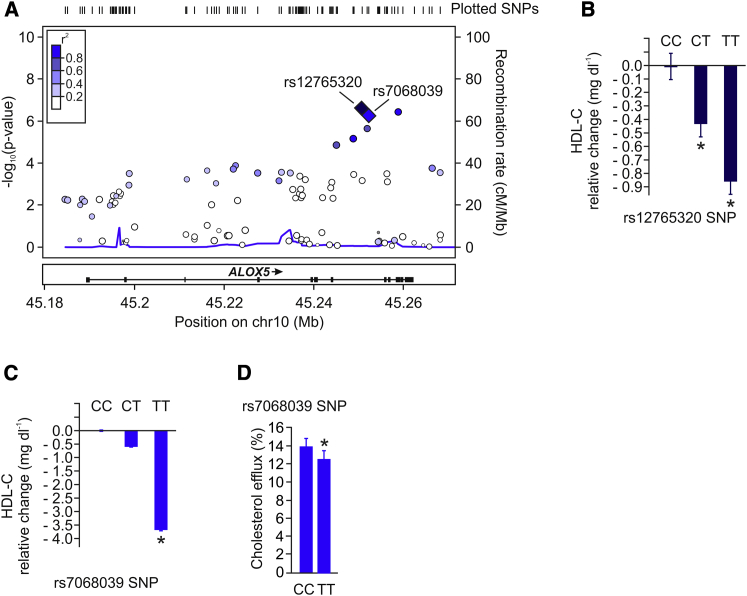
Identification of ALOX5 as Regulator of HDL-C in Humans Common variants in the human *ALOX5* gene were evaluated in a GWAS meta-analysis comprising >100,000 individuals of European ancestry. (A) Manhattan plot for GWA to HDL-C in the *ALOX5* gene, with values of –log_10_ p plotted against position on chromosome 10; colors indicate amount of linkage disequilibrium between SNPs; diamonds indicate the identified lead SNPs rs12765320 and rs7068039 within *ALOX5*. Plots were generated using LocusZoom. (B) Per allele HDL-C relative change in carriers of the lead *ALOX5* SNP rs12765320 as derived from the GLGC data set. (C) Per allele HDL-C relative change in carriers of the lead *ALOX5* SNP rs7068039 in the PLIC replication cohort. (D) Cholesterol efflux using apoB-depleted serum from 30 CC and 30 TT age- and sex-matched rs7068039. Graphs show mean ± SEM, ^∗^p < 0.05.

We first mined published genome-wide association data for signals in and around genes encoding enzymes involved in the metabolism of AA. Using the recently published data of 46 GWASs on the relevance of novel genetic loci for blood lipids in >100,000 individuals of European ancestry (Teslovich et al., 2010), we evaluated whether common variants in the human cyclooxygenases I and II (*PTGS1* and *PTGS2*), *ALOX5*, *ALOX12*, and the *ALOX15* gene loci were associated with alterations in plasma cholesterol levels. We found no association of plasma cholesterol levels to variants within or around the *PTGS1*, *PTGS2*, *ALOX12*, and *ALOX15* genes (data available upon request), whereas robust association signals were observed to variants within the chromosome 10 locus (10q11.21) containing both the *ALOX5* and *MARCH8* genes (see Figure S1 available online), which was confirmed in the 2013 GLGC data set comprising >188,000 individuals (Willer et al., 2013) (Figure S2). Figure 1A shows the signals over the *ALOX5* gene associated with HDL-C, with the ten most significant SNPs within the gene listed in Table S1. No significant associations with LDL-C were observed at this locus, and signals for total cholesterol seem driven by the HDL-C associations. Of note, individuals carrying the common T allele (allele frequency of 0.65) of lead SNP rs12765320 within the *ALOX5* gene showed a dose-dependent decrease in plasma HDL-C levels (ES = −0.429 mg dl^−1^ per copy of T allele; Figure 1B). The reported association of rs12765320 with HDL-C in the GWAS study was independently replicated in the smaller LUdwigshafen RIsk and Cardiovascular Health (LURIC) (Winkelmann et al., 2001) cohort comprising 2,095 individuals (HDL-C 37.23 ± 10.69 mg dl^−1^ versus 36.32 ± 9.42 mg dl^−1^, male homozygous carriers of the CC and the TT alleles, respectively; p < 0.05).

To further support the association between *ALOX5* and HDL-C, a second SNP (rs7068039) belonging to the haplotype block (Table S1) was genotyped in 2,141 individuals from the PLIC (Progressione Della Lesione Intimale Carotidea) study (Norata et al., 2010). Homozygous carriers of the common TT allele presented significantly lower levels of HDL-C compared to homozygous CC carriers (55.11 ± 14.87 mg dl^−1^ versus 58.78 ± 13.88 mg dl^−1^, respectively; p < 0.05) (Figure 1C), thus replicating this association in a second independent cohort. Next, we characterized whether the differences in HDL-C levels also translate into alterations of HDL function. Therefore, cholesterol efflux capacity of apoB- depleted serum from age- and sex-matched TT and CC rs7068039 carriers was measured. Homozygous TT allele carriers showed significantly reduced cholesterol efflux capacity compared to CC carriers (Figure 1D). In summary, GWAS screening in humans helped to identify associations between variants within *ALOX5* not only with HDL-C mass but importantly also with HDL function.

### Aspirin Treatment Promotes RCT

From the GWAS data it cannot be inferred whether the newly identified variants may regulate expression of arachidonate lipoxygenases and whether differential activation of arachidonate lipoxygenases may impact plasma cholesterol levels or affect functional properties of lipoproteins such as RCT. Therefore, we performed functional validation studies in mice using a systematic approach: (1) simultaneous activation of Alox5 and Alox12/15, (2) selective knockout of Alox5, and (3) knockout of Alox12/15. In contrast to humans, who express at least four isoforms of ALOX15, mice do not have separate 12- and 15-arachidonate lipoxygenases, but rather a combined 12/15-lipoxygenase with variable positional specificity for both the 12-position and the 15-position of AA (Kühn and O’Donnell, 2006).

**Figure 2:**
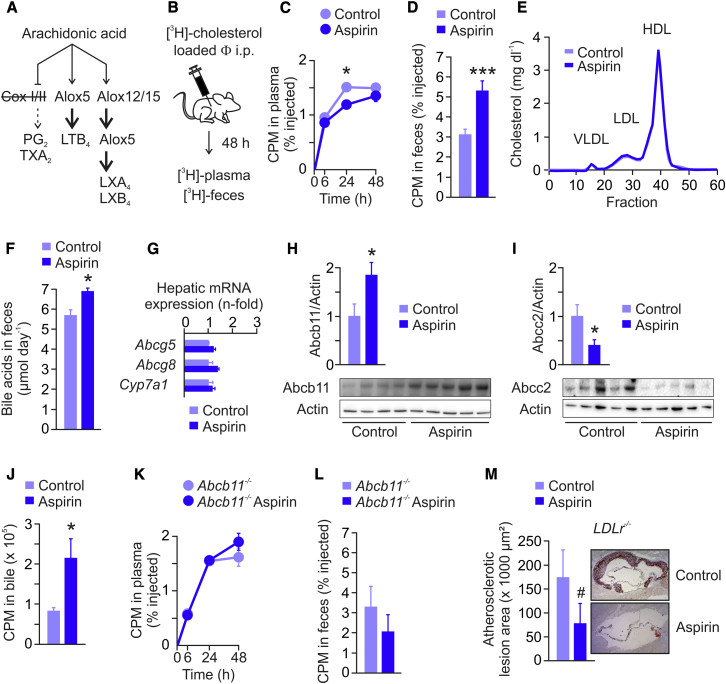
Aspirin Promotes RCT (A) In mice, AA can be metabolized via three main pathways: (1) Cox I/II-mediated generation of prostaglandins and thromboxane (PG_2_, TXA_2_); (2) Alox5-mediated generation of leukotriene B_4_ (LTB_4_) and cysteinyl-leukotrienes; and (3) Alox12/15- and Alox5-mediated generation of lipoxins A_4_ and B_4_ (LXA_4_, LXB_4_). To inhibit Cox I/II, thereby shifting the AA metabolism to Alox5 and Alox12/15 pathways, C57BL/6 mice were treated with aspirin in their drinking water for 7 days. (B) For macrophage-to-feces RCT studies, control and aspirin-treated mice were injected intraperitoneally with cholesterol-loaded, [^3^H]-labeled J774 macrophages (Φ). The tracer was measured in plasma at indicated time points and in fecal sterols collected for 48 hr. (C and D) (C) Plasma [^3^H]-cholesterol levels and (D) fecal [^3^H]-sterol levels (n = 6–10, data representative of three independent macrophage-to-feces RCT experiments). (E) FPLC analysis of plasma pooled from control and aspirin mice (n = 6). (F) Enzymatic measurement of bile acids in feces collected for 48 hr (n = 7). (G) qRT-PCR measurement of neutral sterol transporters *Abcg5* and *Abcg8*, and bile acid converting enzyme *Cyp7a1* in livers of mice (n = 7). (H and I) (H) Immunoblot analysis of bile acid secreting pump Abcb11 and (I) Abcc2 protein expression in livers of mice (n = 4–5, bars represent densitometric quantification normalized to actin). (J) [^14^C]-glycocholic acid was injected into the tail vein of mice, and after 30 min the tracer was quantified in total bile (n = 5). (K) Plasma [^3^H]-cholesterol levels at indicated time-points and (L) fecal [^3^H]-sterol levels (0–48 hr) from a macrophage-to-feces RCT study performed in *Abcb11*^*−/−*^ mice (n = 4–5). (M) Atheroregression in *LDLr*^*−/−*^ mice treated with aspirin. Graphs show mean ± SEM (n = 6), ^#^p = 0.062, ^∗^p < 0.05, ^∗∗∗^p < 0.001.

In our first in vivo experiments, we simultaneously induced the processing of AA by Alox5 and Alox12/15 through pharmacological inhibition of Cox I/II, which shifts the biosynthetic pathways of the AA metabolome toward the formation of LXs and LTs in murine systems (Figure 2A) (Brink et al., 2003, Serhan, 2007, Spite and Serhan, 2010). Inhibition of Cox I/II in mice was achieved by systemic treatment with aspirin (Tancevski et al., 2006), and in vivo macrophage-to-feces RCT studies were performed as described (Tancevski et al., 2010, Zhang et al., 2003): after intraperitoneal injection of [^3^H]-cholesterol-labeled J774 macrophages, the tracer was measured in plasma and feces (Figure 2B). Aspirin-treated mice had significantly decreased plasma [^3^H]-cholesterol levels 24 hr postinjection (Figure 2C), which was associated with significantly increased [^3^H]-sterol levels in feces (Figure 2D). These findings suggested that the increase in fecal tracer content has been caused either by enhanced uptake of [^3^H]-HDL-C into liver and/or by increased biliary transport of sterols. Hepatic protein expression of the HDL receptor (scavenger receptor BI, SR-BI) and of the LDL receptor (LDLr) were unaffected in aspirin-treated mice (Figure S3A), making the hypothesis of enhanced cholesterol clearance from plasma rather unlikely. Accordingly, plasma total cholesterol levels as well as HDL-C levels were unchanged in aspirin-treated mice (Figures S3B and S3C), which was further confirmed by lipoprotein separation analysis via fast protein liquid chromatography (FPLC) (Figure 2E).

### Aspirin Promotes Excretion of Bile Acids

In the liver, a major part of cholesterol is converted into bile acids (Lefebvre et al., 2009), which are secreted into bile for eventual removal via feces (Lefebvre et al., 2009, Stieger, 2009, Zollner and Trauner, 2009). To better define the type of sterols found to be increased in feces of aspirin-treated mice, a subsequent RCT study was performed, and fecal sterols were extracted into neutral and acidic sterols. We found a marked increase in [^3^H]-acidic sterols, i.e., bile acids, underlying the observed increase in total counts (relative content of fecal [^3^H]-acidic sterols, 78% versus 94%, control versus aspirin-treated mice, p < 0.001). Accordingly, in an independent in vivo experiment, Cox I/II inhibition by aspirin was shown to increase the content of fecal bile acid mass per se, as measured by an enzymatic assay (Figure 2F). Plasma levels of 7α-hydroxy-4-cholesten-3-one (C4), a stable plasma marker of bile acid synthesis (Gälman et al., 2003), were not different between aspirin-treated and control mice (Figure S4). In line with unaffected C4 plasma levels, there was no change in hepatic *Cyp7a1* mRNA expression, the rate-limiting enzyme for conversion of cholesterol into bile acids (Figure 2G). Moreover, quantitative real-time PCR with reverse transcription (qRT-PCR) analysis revealed no changes in mRNA levels of hepatic neutral sterol transporters *Abcg5* and *Abcg8* (Figure 2G). Immunoblot analysis showed increased Abcb11 protein levels in livers of aspirin-treated mice (Figure 2H), which constitutes a major determinant of bile flow. On the apical membrane of hepatocytes resides not only Abcb11 but also the multidrug resistance-associated protein 2 (Mrp2 or Abcc2), capable of promoting bile acid excretion (Zollner and Trauner, 2009). Abcb11 mediates excretion of monovalent bile acids, whereas divalent bile acids are exported by Abcc2, which is a conjugate export pump and which has also been discussed as an alternative canalicular bile acid export system in mice (Zollner and Trauner, 2009). In contrast to Abcb11, Abcc2 protein levels were found strongly decreased in livers of aspirin-treated mice (Figure 2I). Under physiological conditions, Abcb11 constitutes the rate-limiting step in bile acid transport from the liver into the bile and subsequently intestine (Figge et al., 2004, Stieger, 2009, Stieger and Beuers, 2011, Zollner and Trauner, 2009), and its transgenic overexpression in mice increases bile flow by 30% and fecal bile acid content by more than 40% (Wang et al., 2010). To firmly establish that Cox I/II inhibition by aspirin increases bile acid excretion, [^14^C]-glycocholic acid was injected into the tail vein of mice, and after 30 min the tracer was quantified in gall bladders of control and aspirin-treated animals (Wang et al., 2001). In confirmation of our data, Cox I/II inhibition by aspirin increased the amount of tracer in bile ∼2-fold (Figure 2J). Finally, treatment of *Abcb11*^*−/−*^ mice with aspirin had no effect on macrophage-to-feces RCT (Figures 2K and 2L), proving the hypothesis of an Abcb11-dependent mechanism. Thus, we conclude that Cox I/II inhibition by aspirin promotes fecal excretion of bile acids and thereby the rate of macrophage-to-feces RCT in mice by increasing hepatic Abcb11 expression.

### Aspirin Induces Regression of Atherosclerosis in *LDLr*^*−/−*^ Mice

Aspirin is one of the most widely used drugs for primary and secondary prevention of CAD, prescribed to patients at high cardiovascular risk (i.e., diagnosed with CAD, diabetics, etc.). So far, the atheroprotective effect of aspirin was related to its antithrombotic and anti-inflammatory/proresolving properties (Spite and Serhan, 2010). However, the identification of aspirin as a RCT-promoting drug also raised the question of whether aspirin could confer regression of established atherosclerosis. For this purpose, we performed a study in male *LDLr*^*−/−*^ mice fed a western-type diet for 14 weeks, then switched to normal chow and divided into two groups, one receiving placebo and the other receiving aspirin in the drinking water for another 6 weeks. Aspirin treatment led to an ∼50% reduction in atherosclerotic lesion size, showing that aspirin can lead to the regression of atherosclerosis (Figure 2M).

### Lipidomic Profiling of Aspirin-Treated Mice

**Figure 3:**
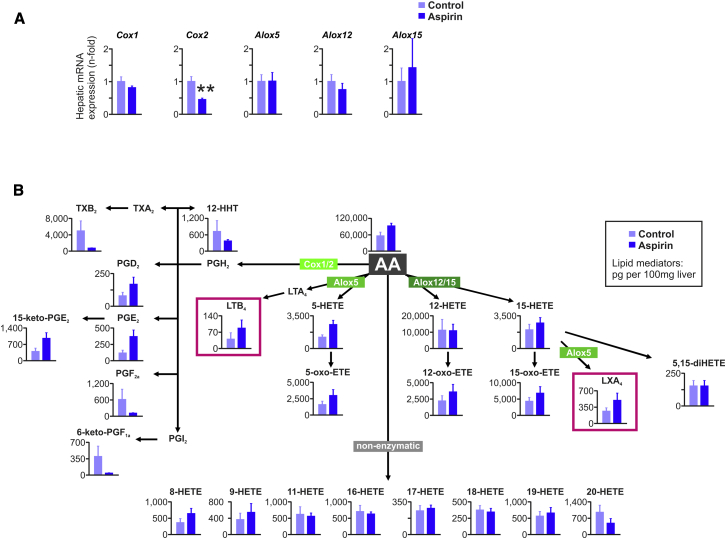
The Impact of Aspirin Treatment on the AA Metabolome (A) qRT-PCR analysis of *Cox1*, *Cox2*, *Alox5*, *Alox12*, *Alox15* in livers of control and of aspirin-treated C57BL/6 mice (n = 6–7), ^∗∗^p < 0.01. (B) Mediator lipidomics in livers of control and of aspirin-treated C57BL/6 mice (n = 5). Graphs show mean ± SEM.

To verify adequate Cox I/II inhibition by aspirin and to analyze changes in intrahepatic levels of LTs and LXs, we next performed qRT-PCR analysis of the genes involved in the biogenesis of these lipids followed by mediator lipidomic analysis (Arita, 2012, Morita et al., 2013). As shown in Figure 3A, and in line with a previous report (Xu et al., 1999), livers of aspirin-treated C57BL/6 mice had reduced *Cox2* expression, whereas the expression of arachidonate lipoxygenases was unaffected by aspirin treatment. Aspirin is expected to inhibit Cox I activity, thereby blocking the formation of prothrombotic TXA_2_. This was confirmed by lipidomic profiling, as levels of the metabolite of TXA_2_, namely TXB_2_, were dramatically reduced in livers of aspirin-treated mice (Figure 3B). Finally, lipidomic profiling revealed that inhibition of the Cox I/II pathway led to enhanced generation of both LTs (LTB_4_) and LXs (LXA_4_) in livers of aspirin-treated animals (Figure 3B). We thus reasoned that the observed increase in hepatic Abcb11 protein expression in mice treated with aspirin may have been conferred by either LTs or LXs, or both.

### Leukotrienes and Lipoxins Regulate Abcb11, SR-BI, and LDLr in Hepatocytes

**Figure 4:**
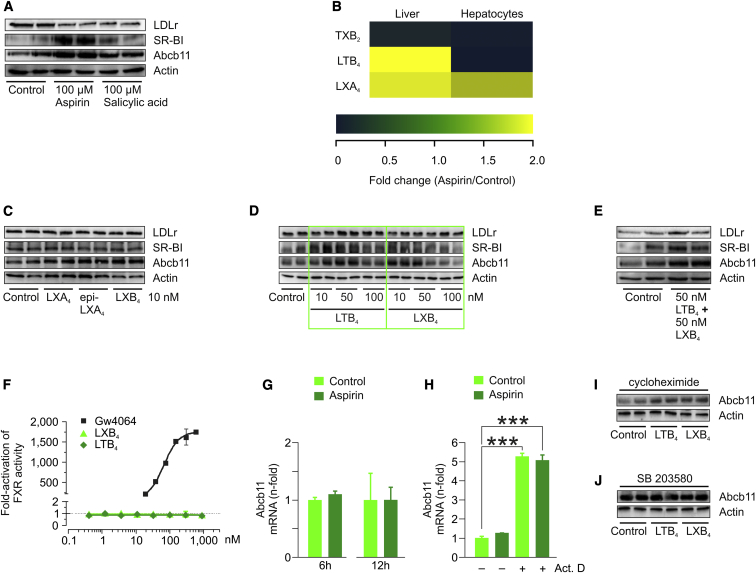
Leukotrienes and Lipoxins Regulate Abcb11 in a Posttranslational Fashion To investigate the mechanisms underlying Abcb11 regulation by aspirin, studies in primary murine hepatocytes derived from C57BL/6 mice were performed. (A and C–E) Hepatocytes were incubated with indicated compounds at given concentrations for 24 hr, after which Abcb11 protein expression was measured by immunoblot analysis. Additionally, protein expression of SR-BI and LDLr was analyzed; actin served as loading control. (B) Heatmap showing relative changes of TXB_2_, LTB_4_, and LXA_4_ levels in livers and hepatocytes measured by means of mediator lipidomics (aspirin/control). (F) FXR reporter assay with positive control (GW4064), LXB_4_, and LTB_4_ used at indicated concentrations. (G) qRT-PCR analysis of *Abcb11* in primary murine hepatocytes treated with vehicle or aspirin (100 μM) for 6 and 12 hr. (H) To measure *Abcb11* mRNA degradation, murine hepatocytes were pretreated with actinomycin D (Act. D), after which they were treated with vehicle or aspirin (100 μM) for 24 hr. RNA levels were measured by qRT-PCR, ^∗∗∗^p < 0.001. (I) Protein translation in murine hepatocytes was inhibited by preincubation with cycloheximide (10 μg ml^−1^) for 6 hr, after which cells were treated with vehicle, LTB_4_ (50 nM), and LXB_4_ (10 nM) for another 18 hr. Immunoblot showing Abcb11 expression; actin served as loading control. (J) Primary murine hepatocytes were coincubated with MAPK p38 ihibitor SB 203580 (10 μM) and vehicle, LTB_4_ (50 nM), and LXB_4_ (10 nM) for 24 hr, after which immunoblot analysis of Abcb11 was performed. Actin served as loading control.

Using primary murine hepatocytes derived from C57BL/6 mice, we confirmed our in vivo studies by demonstrating that both aspirin and its active metabolite salicylic acid induce Abcb11 protein expression. Moreover, aspirin and salicylic acid induced SR-BI protein expression, whereas they downregulated the expression of the LDLr in vitro (Figure 4A). Presently, little is known about lipoxygenase pathways within hepatocytes. One main difference between hepatocytes and other cell types is that hepatocytes do not express Alox5. In this regard, Claría’s lab showed that Kupffer cells, which are of myeloid origin and express high levels of Alox5, are the major source of LTB_4_ and LXs in rat liver exposed to aspirin (Planagumà et al., 2002). Accordingly, when comparing mediator lipidomic profiles of mouse liver and of isolated murine hepatocytes, we found that (1) in both settings, aspirin dramatically reduced TXB_2_ levels as result of efficient Cox I inhibition, (2) in liver extracts, aspirin treatment increased both LTs and LXs, and (3) in isolated mouse hepatocytes, aspirin treatment increased only LX formation (Figure 4B). When analyzing the relative effects of different LX isomers on Abcb11 expression, we found that LXA_4_, 15-epi-LXA_4_, and LXB_4_ increased Abcb11 at 10 nM, with LXB_4_ showing the strongest induction (Figure 4C). Dose-titration studies as well as coincubation experiments with LTB_4_ and LXB_4_ in primary hepatocytes derived from C57BL/6 mice revealed that both lipid mediators increase Abcb11 expression. Intriguingly, LXB_4_ decreased Abcb11 protein expression at high dosages. On the other hand, SR-BI and LDLr protein expression was induced mainly by LTB_4_ (Figures 4D and 4E).

### Leukotrienes and Lipoxins Regulate Abcb11 in a Posttranslational Fashion

To further decipher the molecular mechanisms underlying the induction of Abcb11 by aspirin, we performed further studies in primary hepatocytes. The main positive regulator of Abcb11 expression is the transcription factor farnesoid X receptor (Fxr) (Stieger, 2009, Zollner and Trauner, 2009). However, FXR reporter studies ruled out any direct activation of this nuclear receptor by both LTB_4_ and LXB_4_ (Figure 4F). In line with this finding, LTs and LXs neither induced the transcription of Abcb11 mRNA (Figure 4G), nor did they influence Abcb11 mRNA degradation (Figure 4H). In addition, blocking protein translation by cycloheximide did not abolish Abcb11 protein expression induced by eicosanoids, suggesting that neosynthesis of protein was not required and that a direct regulation was taking place at a posttranslational level (Figure 4I).

Abcb11 protein expression is known to be stabilized by the mitogen-activated protein kinase (MAPK) p38 (Kubitz et al., 2004), and aspirin, in turn, is known to activate p38 in different cell types, including hepatocytes (Oshima et al., 2008, Trujillo-Murillo et al., 2008). As shown in Figure 4J, blockage of p38 by the selective inhibitor SB 203580 abolished the induction of Abcb11 protein expression by LTB_4_ and LXB_4_. To summarize, LTs and LXs increase the expression of Abcb11 by a posttranscriptional and posttranslational mechanism, involving the activity of MAPK p38.

### The Role of Alox5 in Cholesterol Metabolism and RCT

**Figure 5:**
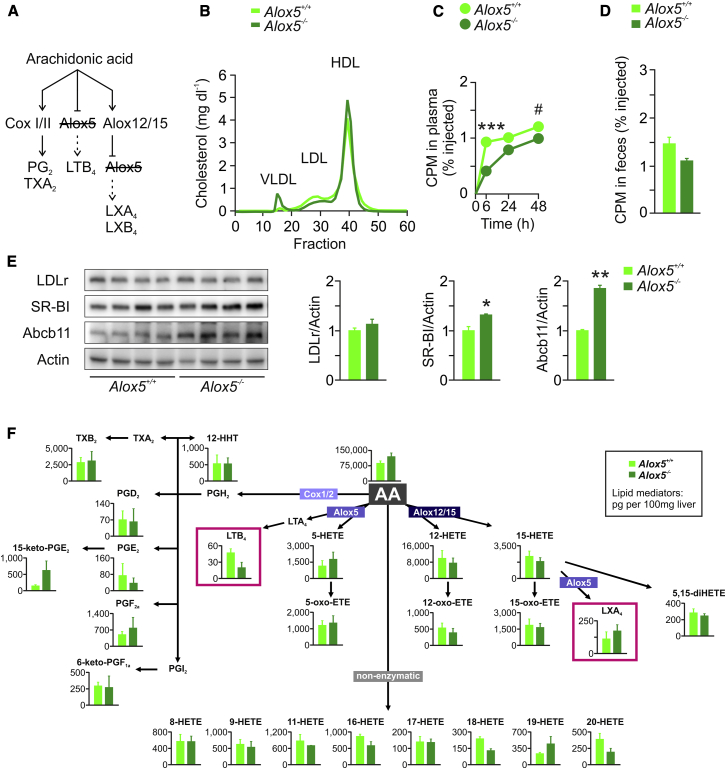
The Role of Alox5 in Abcb11 Regulation and Cholesterol Homeostasis (A) To selectively study the Alox5 pathway, studies in *Alox5*^*+/+*^ and *Alox5*^*−/−*^ mice were performed. (B) FPLC analysis of plasma pooled from *Alox5*^*+/+*^ and *Alox5*^*−/−*^ mice (n = 5). (C) Plasma [^3^H]-cholesterol levels at indicated time points and (D) fecal [^3^H]-sterol levels (0–48 hr) from a macrophage-to-feces RCT study performed in *Alox5*^*+/+*^ and *Alox5*^*−/−*^ mice (n = 3). (E) Immunoblot analysis of LDLr, SR-BI, and Abcb11 protein expression in livers of mice (n = 4; bars represent densitometric quantification normalized to actin). Graphs show mean ± SEM, ^∗^p < 0.05, ^∗∗^p < 0.01, ^∗∗∗^p < 0.001. (F) Mediator lipidomics in livers of *Alox5*^*+/+*^ and *Alox5*^*−/−*^ mice (n = 3). Graphs show mean ± SEM.

Next, we wondered whether knocking out Alox5 would affect hepatic Abcb11 expression, cholesterol homeostasis, and RCT in mice (Figure 5A). As shown in Figure 5B, no marked difference in plasma LDL-C and HDL-C between *Alox5*^*+/+*^ and *Alox5*^*−/−*^ mice was observed, whereas VLDL-C levels increased in the knockouts. In macrophage-to-feces RCT experiments, *Alox5*^*−/−*^ mice showed reduced plasma tracer levels over 48 hr (Figure 5C) but no significant change in fecal excretion of [^3^H]-sterols, when compared to *Alox5*^*+/+*^ mice (Figure 5D). Immunoblot analysis revealed an ∼2-fold induction of hepatic Abcb11 and a moderate increase in SR-BI, but no effect on LDLr protein expression (Figure 5E). Finally, lipidomic profiling of livers from *Alox5*^*+/+*^ and *Alox5*^*−/−*^ mice showed no changes in LX levels, whereas LTB_4_ levels were drastically reduced (Figure 5F).

### The Role of Alox12/15 in Cholesterol Metabolism and RCT

**Figure 6:**
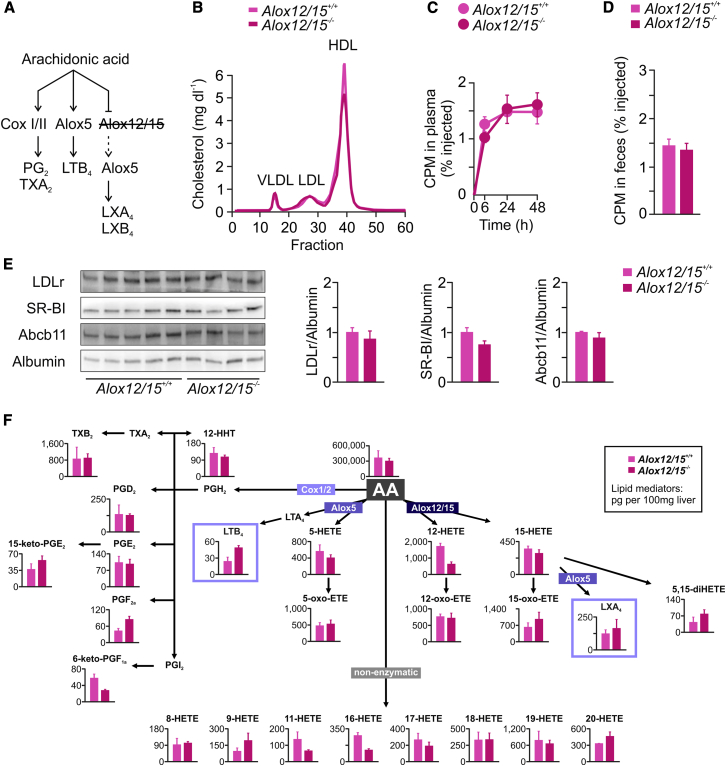
The Role of Alox12/15 in Abcb11 Regulation and Cholesterol Homeostasis (A) To selectively study the Alox12/15 pathway, studies in *Alox12/15*^*+/+*^ and *Alox12/15*^*−/−*^ mice were performed. (B) FPLC analysis of plasma pooled from *Alox12/15*^*+/+*^ and *Alox12/15*^*−/−*^ mice (n = 5). (C) Plasma [^3^H]-cholesterol levels at indicated time points and (D) fecal [^3^H]-sterol levels (0–48 hr) from a macrophage-to-feces RCT study performed in *Alox12/15*^*+/+*^ and *Alox12/15*^*−/−*^ mice (n = 5). (E) Immunoblot analysis of LDLr, SR-BI, and Abcb11 protein expression in livers of mice (n = 4–5; bars represent densitometric quantification normalized to albumin). Graphs show mean ± SEM. (F) Mediator lipidomics in livers of *Alox12/15*^*+/+*^ and *Alox12/15*^*−/−*^ mice (n = 3). Graphs show mean ± SEM.

Lack of Alox12/15 in *Alox12/15*^*−/−*^ mice neither affected plasma cholesterol levels nor influenced macrophage-to-feces RCT, when compared to *Alox12/15*^*+/+*^ mice (Figures 6A–6D). Accordingly, no change in expression of hepatic Abcb11, LDLr, and SR-BI was observed in *Alox12/15*^*−/−*^ mice (Figure 6E). Mediator lipidomic analysis showed unchanged levels of LXs in livers of *Alox12/15*^*−/−*^ mice compared to *Alox12/15*^*+/+*^ mice, whereas LTB_4_ levels were increased in the knockouts (Figure 6F).

### Treatment with Lipoxin Mimetics Lowers Plasma LDL-C

**Figure 7:**
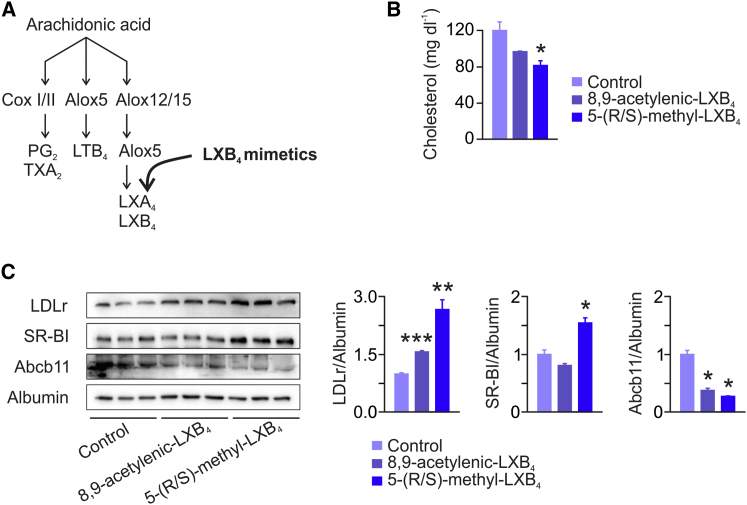
Treatment with Lipoxin Mimetics Lowers Plasma LDL-C (A) C57BL/6 mice were daily injected i.v. with vehicle (control), 10 ng 8,9-acetylenic-LXB_4_, or 10 ng 5-(R/S)-methyl-LXB_4_ for 4 days (data presented are representative of three independent experiments). (B) Plasma total cholesterol measurement in control and 8,9-acetylenic-LXB_4_- and 5-(R/S)-methyl-LXB_4_-treated mice (n = 3). (C) Immunoblot analysis of LDLr, SR-BI, and Abcb11 protein expression in livers of mice (n = 3; bars represent densitometric quantification normalized to albumin). All bars show mean ± SEM, ^∗^p < 0.05, ^∗∗^p < 0.01, ^∗∗∗^p < 0.001.

Finally, in an approach to translate our combined findings from GWAS analysis in humans, mediator lipidomics and functional studies in mice into the identification of novel compounds which beneficially influence plasma cholesterol levels and thus putatively the course of CAD, we reasoned to study the impact of systemic treatment with LXB_4_ on cholesterol metabolism in mice (Figure 7A). Because LXB_4_ is unstable and rapidly inactivated within the circulation, we performed daily i.v. injections into mice with synthetic stable analogs of LXB_4_ including 5-(R/S)-methyl-LXB_4_ and 8,9-acetylenic-LXB_4_, the latter being a log order of magnitude less potent than LXB_4_ (Maddox et al., 1998). After 4 days of treatment, 8,9-acetylenic-LXB_4_ caused a nonsignificant ∼15% decrease in plasma total cholesterol, whereas 5-(R/S)-methyl-LXB_4_ significantly reduced plasma cholesterol levels by ∼30% (Figure 7B). FPLC analysis showed a decrease in LDL-C in 5-(R/S)-methyl-LXB_4_-treated mice, whereas HDL-C remained unchanged (Figure S5). Accordingly, treatment with 5-(R/S)-methyl-LXB_4_ was associated with an ∼3-fold increase in the protein expression of hepatic LDLr and a moderate increase in hepatic protein levels of SR-BI (Figure 7C). Abcb11 protein expression was decreased in animals treated with LX mimetics compared to controls.

## Discussion

Lipoxygenases are lipid peroxidizing enzymes belonging to the nonheme iron dioxygenases family and are conserved across species including bacteria, algae, plants, fish, amphibia, reptilia, and mammals. In animals, the conventional nomenclature classifies lipoxygenases according to their positional specificity of AA oxygenation (Ivanov et al., 2010). Human arachidonate 5-lipoxygenase thus catalyzes oxidation of AA at the 5-position, leading to the formation of hydroxyeicosatetraenoic acids, which can be further metabolized into LTs and LXs through cell-cell interaction. Leukocytes—well-appreciated players in atherosclerosis and myocardial infarction (Drechsler et al., 2010, Dutta et al., 2012)—represent a main source of ALOX5-derived lipid mediators under pathophysiological conditions such as sustained inflammation in atherosclerosis (Spite and Serhan, 2010). Thus *ALOX5* is believed to potentially accelerate atherosclerosis by promoting the inflammatory process within the arterial wall through increased synthesis of LTs by leukocytes (Peters-Golden and Henderson, 2007). Here, we extend this knowledge by showing that *ALOX5* SNPs associate with HDL-C mass and function in human plasma. During the last decade, the old paradigm focusing solely on HDL-C levels as predictor of atherosclerosis changed to a more comprehensive, functional view of HDL particles. In this regard, it was shown that one key function of HDL particles, namely cholesterol efflux capacity from macrophages, has a strong inverse association with atherosclerosis that is independent of HDL-C levels (Khera et al., 2011). Simultaneously, macrophage-to-feces RCT became a recognized key function of HDL important for regression of atherosclerosis (Cuchel and Rader, 2006). Rader and others identified a multitude of novel approaches to promote RCT, including transgenic overexpression of SR-BI or apo-AI, apo-AI-directed therapeutics, and Lxr and Pparα activation (Rosenson et al., 2012). Moreover, biliary sterol secretion was recognized to be required for functional macrophage-to-feces RCT (Nijstad et al., 2011). Here, we show that aspirin, one of the most widely used drugs for prevention of atherosclerosis, promotes macrophage-to-feces RCT by increasing fecal excretion of bile acids. Moreover, we found that aspirin induces regression of established atherosclerosis in mice, which may—besides its well-known anti-inflammatory and platelet-inhibitory effects—be at least in part due to promotion of RCT.

We identify Abcb11 to be upregulated upon aspirin treatment. Analysis of bile acid kinetics, together with macrophage-to-feces RCT measurement in *Abcb11*^*−/−*^ mice, showed that promotion of bile flow is the driving force of RCT in mice treated with aspirin. Failure of aspirin to increase RCT in *Abcb11*^*−/−*^ mice is conceivably due to the lack of Abcb11, which, however, cannot be definitively ruled out, because there may be other hypothetic compensatory mechanisms. Intriguingly, by the help of lipid mediator profiling we found that both Alox5-dependent classes of lipids, namely LTs and LXs, induce protein expression of Abcb11 in a posttranslational fashion, presumably by stabilizing MAPK p38-dependent trafficking of Abcb11 from the Golgi to the cytosol and plasma membrane (Kubitz et al., 2004). Depletion of Kupffer cells by injection of mice with clodronate liposomes abrogated the effect of aspirin on hepatic Abcb11 expression, indicating that these cells serve as a major source for LT and LX formation in vivo (Figure S6). So far, Alox5 was thought to be critical for LT and LX generation. However, our lipidomic analyses in primary murine hepatocytes and in livers of *Alox5*^*−/−*^ mice indicate that in the absence of Alox5 other enzymes may compensate the lack of this lipoxygenase. Interestingly, this alternative pathway remains aspirin responsive, since treatment of *Alox5*^*−/−*^ mice with aspirin led to a further decrease in LT levels (Figure S7), thus indicating the existence of a regulated compensatory mechanism of LT and LX synthesis in liver of mice.

In an effort to better understand the relative contributions of the main lipoxygenases involved in LT and LX synthesis to RCT in mice, we performed a series of loss-of-function experiments. Studies in mice lacking Alox5 or Alox12/15 identified Alox5 as the lipoxygenase with RCT-modifying properties. By combining our mechanistic in vitro experiments with mediator lipidomic profiling, our data suggest that in vivo the balance between LXs and LTs modulates the expression of hepatic Abcb11, i.e., when LX levels exceed LT levels. Surprisingly, *Alox5*^*−/−*^ mice did not show increased excretion of macrophage-derived sterols, despite reduced plasma [^3^H]-cholesterol levels. This may be due to lower biliary excretion of neutral sterols via Abcg5 and Abcg8 and/or increased intestinal absorption of neutral sterols in *Alox5*^*−/−*^ mice, a prediction which will require further studies to be clarified.

Finally, we tested LX mimetics in vivo for the following reasons: first, LX mimetics are stable in circulation and may thus modulate hepatic expression of sterol receptors/transporters in a favorable way; second, LXs have a proresolving function in atherosclerosis. Taken together, these properties may confer atheroprotective effects on LX mimetics. We found that stable LXB_4_ analogs strongly induced hepatic expression of LDLr and accordingly reduced plasma cholesterol levels. To better understand how lipoxin mimetics regulate LDLr expression in liver of mice, further studies aimed at investigating the regulation of known LDLr-modifying machineries including HMG-CoAr-, PCSK9-, and IDOL-dependent pathways, together with the analysis of cholesterol-modulating miRNAs in livers of LX-treated mice, are needed. Intriguingly, we found that Abcb11 protein levels were reduced in livers of mice treated with LX mimetics. From our lipidomic analyses we learned that in vivo Abcb11 can be induced when (1) both LTs and LXs are increased, and when (2) LXs remain unchanged and LTs are reduced, whereas Abcb11 expression is unaltered when LXs remain unchanged and LTs increase. These data indicate that in the liver LX generation is tightly regulated and that LXs are probably a more important regulator of Abcb11 than LTs. In fact, our in vitro experiments showed that LXs induce Abcb11 only at low concentrations, whereas they decrease its expression when given at higher dosages. We thus conclude that LXs may regulate Abcb11 with higher specificity than LTs and speculate that exogenously administered LXs could have critically altered lipid mediator homeostasis in the liver, thereby decreasing Abcb11 expression.

Our results support the notion that modulation of the AA metabolome may be used to treat and prevent CAD, and may explain how omega-6 PUFAs influence cholesterol homeostasis. In humans, most PUFAs in the diet consist of the omega-6 type including linoleic acid which is converted into the metabolically important AA after consumption (Harris et al., 2009, Katan, 2009). Higher omega-6 PUFA levels were shown to improve insulin resistance, to reduce the incidence of diabetes mellitus, and to associate with lower blood pressure. Moreover, omega-6 PUFAs were shown to lower plasma LDL-C and plasma total cholesterol-to-HDL-C ratio (summarized in Harris et al., 2009). These effects are believed to confer omega-6 PUFAs and especially AA with atheroprotective properties (Harris et al., 2009, Katan, 2009). Combined data from randomized trials, case-control and cohort studies, and animal experiments indicate that the consumption of at least 5%–10% of energy from omega-6 PUFAs reduces the cardiovascular risk (Harris et al., 2009), which recently prompted the American Heart Association to release a recommendation for dietary supplementation with omega-6 PUFAs (Harris et al., 2009). Using a systematic, interdisciplinary approach, we were able to elucidate the relative role of key players of the AA metabolism in whole-body cholesterol homeostasis in humans and in mice.

To summarize, we show that pharmacological and genetic modulation of the AA metabolome affects one major function of HDL, namely RCT. Moreover, we show that the AA metabolome is a conserved regulator of HDL-C in humans and in mice, and identify LX mimetics (Maddox et al., 1998) as an approach to reduce plasma LDL-C. However, it is appropriate to issue certain caveats when trying to extrapolate mouse data to humans, since mouse models have limitations for the following reasons: mice have a different lipoprotein profile when compared to humans, with HDL being the major lipoprotein fraction in plasma; they do not express cholesteryl ester transfer protein in plasma; and they do not develop atherosclerosis when fed a high-cholesterol diet. Although we cannot directly extrapolate our data on Abcb11, SR-BI, and LDLr to human cholesterol metabolism, we definitively show that the AA metabolome plays a physiological role in whole-body cholesterol homeostasis and HDL function in mammals, paving the way for the development of novel lipid-lowering drugs based on the structure of AA metabolites and offering a novel therapeutic strategy to counteract CAD in humans.

## Experimental Procedures

### Human Genome-Wide Association Data

Genome-wide association data with HDL-C, LDL-C, and total cholesterol was generated by the Global Lipids Genetics Consortium (GLGC) as previously described (Teslovich et al., 2010): http://www.sph.umich.edu/csg/abecasis/public/lipids2010/. The GLGC 2013 data set was recently published (Willer et al., 2013): http://www.sph.umich.edu/csg/abecasis/public/lipids2013/. Plots used in Figures 1, S1, and S2 were generated using LocusZoom (Pruim et al., 2010).

### Animal Studies

All animals were handled in strict accordance with good animal practice as defined by the Austrian Authorities, and all animal work was approved by the Austrian Animal Care and Use Committee (Bundesministerium für Wissenschaft und Forschung–BMWF). Mice were fed a standard chow diet (Ssniff). To induce Cox I/II inhibition, mice were treated with drinking water containing aspirin for 7 days, as described previously by our laboratory (Tancevski et al., 2006). On a body-scale-adjusted scale, the amount of aspirin would be equal to ∼360 mg per day if the animals weighed 60 kg (6 mg kg^−1^ per day) (Tancevski et al., 2006).

### Mediator Lipidomics

LC-MS/MS-based lipidomic analyses were performed using a high-performance liquid chromatography (HPLC) system (Waters UPLC) with a linear ion trap quadrupole mass spectrometer (QTRAP5500; AB SCIEX) equipped with an Acquity UPLC BEH C_18_ column (Waters) as described (Arita, 2012, Morita et al., 2013). MS/MS analyses were conducted in negative ion mode, and fatty acid metabolites were identified and quantified by multiple reaction monitoring (MRM). Different tissue isolation procedures and/or different age or body weight of mice could cause differences in the basal levels of AA metabolites. Thus, lipid mediator levels shown in Figures 3B, 5F, 6F, and S7 cannot be directly compared.

### Statistical Analysis

Statistical analysis was carried out with a SPSS statistical package (IBM). We determined significance by unpaired two-tailed Student’s t test, or by one-way ANOVA when more than two groups were compared. p < 0.05 was considered statistically significant.

## Author Contributions

I. Tancevski conceived the study. E.D., A.S., K.A., C.H., J.R.P., P.E., M.T., I. Theurl, M. Theurl, M.S., D.L., U.S., D.H., M.A., S.D., M.N., E.H., M.S., A.R.M., X.L., P.P., H.S., T.S., W.M., M.E.K, K.G., P.U., A.L.C., F.S., M.R., K.K., Y.I., M.A., J.D.S., P.P.P., U.J.F.T., M. Trauner., G.D.N., T.C., A.A.H., G.W., and I. Tancevski performed the experiments and analyzed and interpreted the data. I. Tancevski, P.E., U.J.F.T, G.D.N., A.A.H., T.C., M. Trauner, and G.W. wrote the paper.

## Supplemental Information

Document S1. Supplemental Experimental Procedures, Tables S1 and S2, and Figures S1–S7

Document S2. Article plus Supplemental Information
