# Supplementary material for: The Arachidonic Acid Metabolome Serves as a Conserved Regulator of Cholesterol Metabolism
Source: Cell Metab. 2014 Nov 4;20(5):787–98. doi: 10.1016/j.cmet.2014.09.004 (PMC4232508; doi:10.1016/j.cmet.2014.09.004)
Supplement: Document S1. Supplemental Experimental Procedures, Tables S1 and S2, and Figures S1–S7 [file mmc1.pdf]

Cell Metabolism, Volume *20*

Supplemental Information

The Arachidonic Acid Metabolome Serves as a Conserved Regulator of Cholesterol Metabolism

Egon Demetz, Andrea Schroll, Kristina Auer, Christiane Heim, Josef R. Patsch, Philipp Eller, Markus Theurl, Igor Theurl, Milan Theurl, Markus Seifert, Daniela Lener, Ursula Stanzl, David Haschka, Malte Asshoff, Stefanie Dichtl, Manfred Nairz, Eva Huber, Martin Stadlinger, Alexander R. Moschen, Xiaorong Li, Petra Pallweber, Hubert Scharnagl, Tatjana Stojakovic, Winfried März, Marcus E. Kleber, Katia Garlaschelli, Patrizia Uboldi, Alberico L. Catapano, Frans Stellaard, Mats Rudling, Keiji Kuba, Yumiko Imai, Makoto Arita, John D. Schuetz, Peter P. Pramstaller, Uwe J.F. Tietge, Michael Trauner, Giuseppe D. Norata, Thierry Claudel, Andrew A. Hicks, Guenter Weiss, and Ivan Tancevski

Supplemental Information

The Arachidonic Acid Metabolome Serves as a Conserved Regulator of Cholesterol Metabolism

Egon Demetz, Andrea Schroll, Kristina Auer, Christiane Heim, Josef R. Patsch, Philipp Eller, Markus Theurl, Igor Theurl, Milan Theurl, Daniela Lener, Ursula Stanzl, Markus Seifert, David Haschka, Malte Asshoff, Stefanie Dichtl, Manfred Nairz, Eva Huber, Martin Stadlinger, Alexander R. Moschen, Xiaorong Li, Petra Pallweber, Hubert Scharnagl, Tatjana Stojakovic, Winfried März, Marcus E. Kleber, Katia Garlaschelli, Patrizia Uboldi, Alberico L. Catapano, Frans Stellaard, Mats Rudling, Keiji Kuba, Yumiko Imai, Makoto Arita, John D. Schuetz, Peter P. Pramstaller, Uwe J. F. Tietge, Michael Trauner, Giuseppe D. Norata, Thierry Claudel, Andrew A. Hicks, Guenter Weiss, and Ivan Tancevski

SUPPLEMENTAL EXPERIMENTAL PROCEDURES

Chemicals and Reagents

Aspirin, sodium salicylate, SB 203580, cycloheximide, GW4064 (Sigma), LTB_4, 15-epi-LXA_4, LXA_4 and LXB_4 (Cayman Chemicals), [^{3}H]-cholesterol (NET139001MC, PerkinElmer) and [^{14}C]-glycocholic acid (NEC838050UC, PerkinElmer). 5-(R/S)-methyl-LXB_4 and 8,9-acetylenic-LXB_4 were a kind gift from Professor Charles N. Serhan, Brigham and Women's Hospital, Harvard University, Boston, MA (Maddox et al., 1998).

Animal Studies

At the end of the studies, animals were fasted for 4 h and anesthetized. Blood samples were taken, mice sacrificed by cervical dislocation, and liver biopsies were snap-frozen. Kupffer cell depletion in mice was induced via iv-injection of clodronate-liposomes as described previously by our laboratory (Theurl et al., 2008). For atherosclerosis studies, male *LDLr*$^{-/-}$ mice (Jackson Laboratories Germany) were fed a Western-type diet for 14 weeks, then switched to normal chow and treated either with water containing aspirin (6 mg kg^{-1} per d) or vehicle for another 6 weeks. At the end of the study, lipoprotein separation of pooled plasma revealed a 50% reduction of HDL-C in the aspirin treated group (114.9 vs. 68.0 mg dl^{-1}, control vs. aspirin). Atherosclerosis lesion area was quantified as described previously (Tancevski et al., 2010). Male C57BL/6, *Alox5*$^{+/+}$, and *Alox5*$^{-/-}$ mice were obtained from

Jackson Laboratories Germany. Male *Alox12/15*$^{+/+}$and *Alox12/15*$^{-/-}$ mice obtained from Jackson Laboratories were bred at Akita University, Japan and University of Groningen, The Netherlands. Male *Abcb11*$^{-/-}$ on C57BL/6 background were described previously (Zhang et al., 2012). Of note, *Abcb11*$^{-/-}$ mice displayed similar plasma HDL-C levels compared to littermate controls (56.8 ± 1.5 vs. 57.2 ± 10.0 mg dl^{-1}, *Abcb11*$^{+/+}$ vs. *Abcb11*$^{-/-}$ mice, n = 5, mean ± SEM, n.s.).

Macrophage-to-feces RCT Studies

Macrophage *in vivo* RCT studies using J774 macrophages (ATCC) were performed as described (Naik et al., 2006; Tancevski et al., 2010; Zhang et al., 2003). J774 macrophages were grown in suspension using a CELLspin 500 (Integra Biosciences), radiolabeled with 2.5 µCi ml^{-1} [^{3}H]-cholesterol and loaded with 40 µg ml^{-1} acetylated LDL (AcLDL) for 48 h. These foam cells were washed twice, equilibrated in medium with 0.2% bovine serum albumin for 6 h, spun down, and resuspended in PBS. [^{3}H]-cholesterol–labeled and AcLDL-loaded J774 cells (typically 5×10^6 cells containing 2×10^6 counts per minute [CPM] in 0.6 ml PBS) were injected intraperitoneally (Tancevski et al., 2010). Plasma samples were taken at 6, 24, and 48 h post-injection. Feces were collected continuously from 0 to 48 h and stored at 4°C before extraction of sterols (Naik et al., 2006). At study termination (48 h after injection), mice were exsanguinated. Fecal cholesterol as well as bile acid extractions were performed as described (Naik et al., 2006). Radioactivity in plasma and in fecal lipid extracts was measured in a liquid scintillation counter, and is given as % CPM injected.

[^{14}C]-glycocholic Acid Study

To determine the bile flow, a previously described protocol was modified (Wang et al., 2001): Trace amounts of [^{14}C]-glycocholic acid were injected into the tail vein of mice, and after 30 minutes the tracer was quantified in total bile of mice using a liquid scintillation counter.

Plasma and Fecal Sterol Analysis

Total cholesterol and PEGylated HDL-C in mouse plasma were measured using a Roche commercial kit as described (Tancevski et al., 2010). Additionally, in some experiments pooled plasma from each group was subjected to FPLC fractionation analysis with two tandem Superose 6 columns (GE Healthcare) as described previously, and cholesterol was measured in single fractions employing a Roche commercial kit (Tancevski et al., 2010). Fecal bile acid mass analysis and measurement of

plasma 7α-hydroxy-4-cholesten-3-one (C4) were performed as described (Galman et al., 2003; Tancevski et al., 2010).

Studies in Primary Murine Hepatocytes

Primary murine hepatocytes were prepared as described (Theurl et al., 2008). Hepatocytes were treated *in vitro* with indicated reagents or vehicle (DMSO) in serum-free medium for 24 h, after which protein or RNA extraction was performed. For the actinomycin D and cycloheximide experiments, cells were pre-treated before adding indicated compounds as described (Kubitz et al., 2004; Sirvent et al., 2004).

FXR reporter assay

FXR reporter assay was performed by Indigo Biosciences, State College, PA, USA.

Protein Extraction and Immunoblot Analysis

Preparation of proteins and subsequent immunoblot analysis were performed as described (Tancevski et al., 2010). The anti-Abcb11 antibody was previously described by Renxue Wang (Wang et al., 2001). Anti-Abcc2, anti-LDLr and anti-Albumin antibodies were from ABCAM, anti-SR-BI was from Novus Biologicals, anti-Actin from Sigma Aldrich. The chemoluminescent reaction was performed using Super Signal West Dura Reagent (Pierce), blots were visualized by Fluor-S-Imager using Quantity One V4.1 software (BioRad) (Tancevski et al., 2010). Densitometric quantification was performed by the use of ImageJ.

RNA Isolation, Reverse Transcription, and Quantitative Real-time PCR (qRT-PCR)

Total RNA was extracted using RNA bee according to the manufacturer's protocol (Tel-test Inc) and reverse transcribed with Omniscript-RT Kit (Qiagen). Primers are as follows:

Abcb11: 5’ ATT GAA CTC CCC ATC GAG CC 3’, 5‘ GCT GGG ATA TGC TTG GCA TTG 3’;

Cyp7a1: 5’ TCT CTG AAG CCA TGA TGC AAA 3’, 5‘ TGA CCC AGA CAG CGC TCT T 3’;

Abcg5: 5’ ATT ATG TGC ATC TTA GGC AGC TC 3’, 5‘ CGT AGG AGA AGC AGT CTT GGA A 3’;

Abcg8: 5’ AGT GGT CAG TCC AAC ACT CTG 3’, 5‘ GAG ACC TCC AGG GTA TCT TGA A 3’;

Rps29: 5’ AGG ACA TAG GCT TCA TTA AGT TGG 3’; 5’ AGC ATG ATC GGT TCC ACT TG 3’;

Alox5: 5’ GGA ACT GCA GGA CTT CGT GA 3’; 5’ CCT GCT CTT GAT GGA CTT GG 3’;

Alox12: 5' GGA CAA GTG CAG AGG CCG TGT T 3'; 5' CAT TGT CTC CTG CCA GGC GG 3';

Alox15: 5' ACA CTT GGT GGC TGA GGT CTT TG 3'; 5' CGG ACA TTG ATT TCC ATG GTG TAG 3';

Cox1: 5' CCA GAA CCA GGG TGT CTG TGT C 3'; 5' ACA GTT GGG GCC TGA GTA GC 3';

Cox2: 5' ACC CAT CAG TTT TTC AAG ACA GAT 3'; 5' GCG CAG TTT ATG TTG TCT GT 3';

Real-time PCR reactions were performed on a BioRad C1000 Cycler.

Replication Cohorts

The LUdwigshafen RIsk and Cardiovascular Health (LURIC) Study was described previously (Winkelmann et al., 2001). The Progressione Della Lesione Intimale Carotidea (PLIC) study was described previously (Norata et al., 2010; Norata et al., 2009); SNP rs7068039 was genotyped in 2,141 individuals from this cohort, cholesterol efflux using apoB- depleted serum from 30 CC and 30 TT age- and sex-matched rs7068039 carriers was performed as described (Baragetti et al., 2013). Anthropomorphic characteristics and biochemical parameters in the PLIC population according to the *ALOX5* genotype are presented in **Table S2**.

SUPPLEMENTAL TABLES

Table S1, related to Figure 1. Ten Most Significantly HDL-C Associated SNPs within *ALOX5* in the GLGC 2010 Published Dataset

SNP	Chr	Position	A1	A2	*P* value	Effect (mg dl^{-1})	SE	eQTL[a]
rs7918542	10	45216257	A	G	2.3×10^{-4}	-0.271	0.093	NV
rs11239515	10	45222061	A	G	1.9×10^{-4}	-0.278	0.100	NV
rs11239516	10	45222543	T	G	1.3×10^{-4}	0.294	0.100	NV
rs10900215	10	45227460	C	G	2.8×10^{-4}	0.275	0.100	5.8×10^{-5}
rs11239524	10	45232777	T	G	2.7×10^{-4}	-0.271	0.100	3.0×10^{-4}
rs3780908	10	45244974	A	C	1.3×10^{-5}	0.348	0.097	NV
rs1487562	10	45248828	T	C	7.0×10^{-6}	0.336	0.093	NV
**rs12765320**	10	45250811	T	C	**2.8×10^{-7}**	**-0.429**	**0.097**	NV
rs7080474	10	45251905	T	C	2.1×10^{-6}	0.394	0.097	NV
rs7068039	10	45252270	T	C	5.4×10^{-7}	-0.386	0.097	NV

Results for all SNPs are based on the published GLGC data (Teslovich et al., 2010). The most significantly associated SNP within *ALOX5* (rs12765320) in this published data is highlighted. Chr, chromosome; A1, allele 1 (effect allele); A2, allele 2; SE, standard error; NV, non verifiable; eQTL, expression quantitative trait loci; [a]derived from the mRNA by SNP Browser 1.0.1 (2008) (Dixon et al., 2007; Moffatt et al., 2007).

Table S2, related to Figure 1. Anthropomorphic characteristics and biochemical parameters in the PLIC population according to the *ALOX5* genotype

Genotype	**CC (n=30)**	**TT (n=30)**	
	**Mean ± SD**	**Mean ± SD**	***P* value**
Age	55.3 ± 10.5	54.7 ± 10.9	n.s.
BMI (Kg m^{-2})	25.6 ± 3.4	26.3 ± 3.7	n.s.
Waist (cm)	87.1 ± 9.3	88.4 ± 10.2	n.s.
Hip (cm)	102.6 ± 6.5	103.8 ± 7.6	n.s.
Waist/Hip ratio	0.85 ± 0.07	0.86 ± 0.8	n.s.
PAS (mmHg)	125 ± 14	123 ± 16	n.s.
PAD (mmHg)	79 ± 6	80 ± 8	n.s.
TC (mg dl^{-1})	207.2 ± 32	215.8 ± 32.2	n.s.
LDL-C (mg dl^{-1})	127.4 ± 29.4	137.3 ± 29.5	n.s.
HDL-C (mg dl^{-1})	64.6 ± 15.9	59.8 ± 15.8	n.s.
TG (mg dl^{-1})	76.3 ± 31.2	93.6 ± 35.1	n.s.
Glucose (mg dl^{-1})	88 ± 9.9	88.6 ± 9.3	n.s.

Anthropomorphic characteristics and biochemical parameters of the individuals from the PLIC study (Norata et al., 2010) whose serum was used to measure cholesterol efflux capacity according to the

ALOX5 genotype shown in **Figure 1D**. BMI, body mass index; PAS, systolic arterial pressure; PAD, diastolic arterial pressure; TC, total plasma cholesterol; TG, triglycerides.

SUPPLEMENTAL FIGURE LEGENDS

Figure S1, related to Figure 1. Genetic Variants Associated with HDL-C on Chr10 Locus in Humans, GLGC 2010 dataset

Common variants in genes involved in the processing of AA associated with HDL-C were evaluated in a GWAS meta-analysis comprising >100,000 individuals from European Ancestry (Teslovich et al., 2010). Strong association signals were identified on chromosome 10 around the genes coding for *ALOX5*, *MARCH8*, and *ANUBL1*. The Figure shows a Manhattan plot for GWA to HDL-C levels, with values of $-\log_{10}$ *P* plotted against chromosomal position. Colors indicate degree of linkage disequilibrium between variants. Plots were generated using LocusZoom (Pruim et al., 2010).

Figure S2, related to Figure 1. Genetic Variants Associated with HDL-C on Chr10 Locus in Humans, GLGC 2013 dataset

Common variants in genes involved in the processing of AA associated with HDL-C were evaluated in a GWAS meta-analysis comprising >188,000 individuals (Willer et al., 2013). Strong association signals were identified on chromosome 10 around the genes coding for *ALOX5* and *MARCH8*. The Figure shows a Manhattan plot for GWA to HDL-C levels, with values of –log10 P plotted against chromosomal position. Colors indicate degree of linkage disequilibrium between variants. Plots were generated using LocusZoom (Pruim et al., 2010).

Figure S3, related to Figure 2. Cox I/II Inhibition Does not Alter Plasma Cholesterol Levels

(A) Immunoblot analysis of LDLr and SR-BI protein expression in livers, as well as (B) total cholesterol and (C) HDL-C measurement in plasma of control and aspirin-treated C57BL/6 mice (n = 7). Bars show mean ± SEM, no significant statistical differences between control and aspirin-treated mice were found.

Figure S4, related to Figure 2. Cox I/II inhibition Does not Alter Bile Acid Synthesis

Measurement of 7α-hydroxy-4-cholesten-3-one (C4), a stable intermediate of bile acid synthesis, in plasma of control and aspirin-treated C57BL/6 mice (n = 7). Bars show mean ± SEM, no significant statistical differences between control and aspirin-treated mice were found.

Figure S5, related to Figure 7. Treatment with Lipoxin Mimetics Lowers Plasma LDL-C

FPLC analysis of plasma pooled from C57BL/6 mice treated i.v. with vehicle (Control) or 5-(R/S)-methyl-LXB_4 for 4 days (10 ng/day, n = 3).

Figure S6, related to Figure 2. Kupffer cells are the major source of LTs and LXs *in vivo*

Male C57BL/6 mice were treated with aspirin or vehicle in their drinking water for 7 days; control liposomes or clodronate-liposomes were injected via tail vein at days 0 and 3 as indicated. At study termination, hepatic Abcb11 protein expression was determined by western blot analysis, Actin served as loading control (upper panels). Depletion of Kupffer cells was ascertained by immunofluorescence staining of liver sections (Kupffer cells, GFP green, nuclei DAPI blue; lower panels).

Figure S7, related to Figure 5. Mediator lipidomics in livers of *Alox5*$^{-/-}$ mice treated with aspirin

Mediator lipidomics in livers of *Alox5*$^{+/+}$, *Alox5*$^{-/-}$ and aspirin-treated *Alox5*$^{-/-}$ mice (n = 3). Graphs show mean ± SEM.

REFERENCES

Baragetti, A., Norata, G. D., Sarcina, C., Rastelli, F., Grigore, L., Garlaschelli, K., Uboldi, P., Baragetti, I., Pozzi, C., and Catapano, A. L. (2013). High density lipoprotein cholesterol levels are an independent predictor of the progression of chronic kidney disease. J Intern Med.

Dixon, A. L., Liang, L., Moffatt, M. F., Chen, W., Heath, S., Wong, K. C., Taylor, J., Burnett, E., Gut, I., Farrall, M., *et al.* (2007). A genome-wide association study of global gene expression. Nat Genet *39*, 1202-1207.

Galman, C., Arvidsson, I., Angelin, B., and Rudling, M. (2003). Monitoring hepatic cholesterol 7alpha-hydroxylase activity by assay of the stable bile acid intermediate 7alpha-hydroxy-4-cholesten-3-one in peripheral blood. J Lipid Res *44*, 859-866.

Kubitz, R., Sutfels, G., Kuhlkamp, T., Kolling, R., and Haussinger, D. (2004). Trafficking of the bile salt export pump from the Golgi to the canalicular membrane is regulated by the p38 MAP kinase. Gastroenterology *126*, 541-553.

Maddox, J. F., Colgan, S. P., Clish, C. B., Petasis, N. A., Fokin, V. V., and Serhan, C. N. (1998). Lipoxin B4 regulates human monocyte/neutrophil adherence and motility: design of stable lipoxin B4 analogs with increased biologic activity. FASEB J *12*, 487-494.

Moffatt, M. F., Kabesch, M., Liang, L., Dixon, A. L., Strachan, D., Heath, S., Depner, M., von Berg, A., Bufe, A., Rietschel, E., *et al.* (2007). Genetic variants regulating ORMDL3 expression contribute to the risk of childhood asthma. Nature *448*, 470-473.

Naik, S. U., Wang, X., Da Silva, J. S., Jaye, M., Macphee, C. H., Reilly, M. P., Billheimer, J. T., Rothblat, G. H., and Rader, D. J. (2006). Pharmacological activation of liver X receptors promotes reverse cholesterol transport in vivo. Circulation *113*, 90-97.

Norata, G. D., Garlaschelli, K., Grigore, L., Raselli, S., Tramontana, S., Meneghetti, F., Artali, R., Noto, D., Cefalu, A. B., Buccianti, G., *et al.* (2010). Effects of PCSK9 variants on common carotid artery intima media thickness and relation to ApoE alleles. Atherosclerosis *208*, 177-182.

Norata, G. D., Garlaschelli, K., Grigore, L., Tibolla, G., Raselli, S., Redaelli, L., Buccianti, G., and Catapano, A. L. (2009). Circulating soluble receptor for advanced glycation end products is inversely associated with body mass index and waist/hip ratio in the general population. Nutr Metab Cardiovasc Dis *19*, 129-134.

Pruim, R. J., Welch, R. P., Sanna, S., Teslovich, T. M., Chines, P. S., Gliedt, T. P., Boehnke, M., Abecasis, G. R., and Willer, C. J. (2010). LocusZoom: regional visualization of genome-wide association scan results. Bioinformatics *26*, 2336-2337.

Sirvent, A., Claudel, T., Martin, G., Brozek, J., Kosykh, V., Darteil, R., Hum, D. W., Fruchart, J. C., and Staels, B. (2004). The farnesoid X receptor induces very low density lipoprotein receptor gene expression. FEBS Lett *566*, 173-177.

Tancevski, I., Demetz, E., Eller, P., Duwensee, K., Hoefer, J., Heim, C., Stanzl, U., Wehinger, A., Auer, K., Karer, R.*, et al.* (2010). The liver-selective thyromimetic T-0681 influences reverse cholesterol transport and atherosclerosis development in mice. PLoS One *5*, e8722.

Teslovich, T. M., Musunuru, K., Smith, A. V., Edmondson, A. C., Stylianou, I. M., Koseki, M., Pirruccello, J. P., Ripatti, S., Chasman, D. I., Willer, C. J.*, et al.* (2010). Biological, clinical and population relevance of 95 loci for blood lipids. Nature *466*, 707-713.

Theurl, M., Theurl, I., Hochegger, K., Obrist, P., Subramaniam, N., van Rooijen, N., Schuemann, K., and Weiss, G. (2008). Kupffer cells modulate iron homeostasis in mice via regulation of hepcidin expression. J Mol Med (Berl) *86*, 825-835.

Wang, R., Salem, M., Yousef, I. M., Tuchweber, B., Lam, P., Childs, S. J., Helgason, C. D., Ackerley, C., Phillips, M. J., and Ling, V. (2001). Targeted inactivation of sister of P-glycoprotein gene (spgp) in mice results in nonprogressive but persistent intrahepatic cholestasis. Proc Natl Acad Sci U S A *98*, 2011-2016.

Willer, C. J., Schmidt, E. M., Sengupta, S., Peloso, G. M., Gustafsson, S., Kanoni, S., Ganna, A., Chen, J., Buchkovich, M. L., Mora, S., *et al.* (2013). Discovery and refinement of loci associated with lipid levels. Nat Genet *45*, 1274-1283.

Winkelmann, B. R., Marz, W., Boehm, B. O., Zotz, R., Hager, J., Hellstern, P., and Senges, J. (2001). Rationale and design of the LURIC study--a resource for functional genomics, pharmacogenomics and long-term prognosis of cardiovascular disease. Pharmacogenomics *2*, S1-73.

Zhang, Y., Li, F., Patterson, A. D., Wang, Y., Krausz, K. W., Neale, G., Thomas, S., Nachagari, D., Vogel, P., Vore, M., *et al.* (2012). Abcb11 deficiency induces cholestasis coupled to impaired beta-fatty acid oxidation in mice. J Biol Chem *287*, 24784-24794.

Zhang, Y., Zanotti, I., Reilly, M. P., Glick, J. M., Rothblat, G. H., and Rader, D. J. (2003). Overexpression of apolipoprotein A-I promotes reverse transport of cholesterol from macrophages to feces in vivo. Circulation *108*, 661-663.

Figure S1

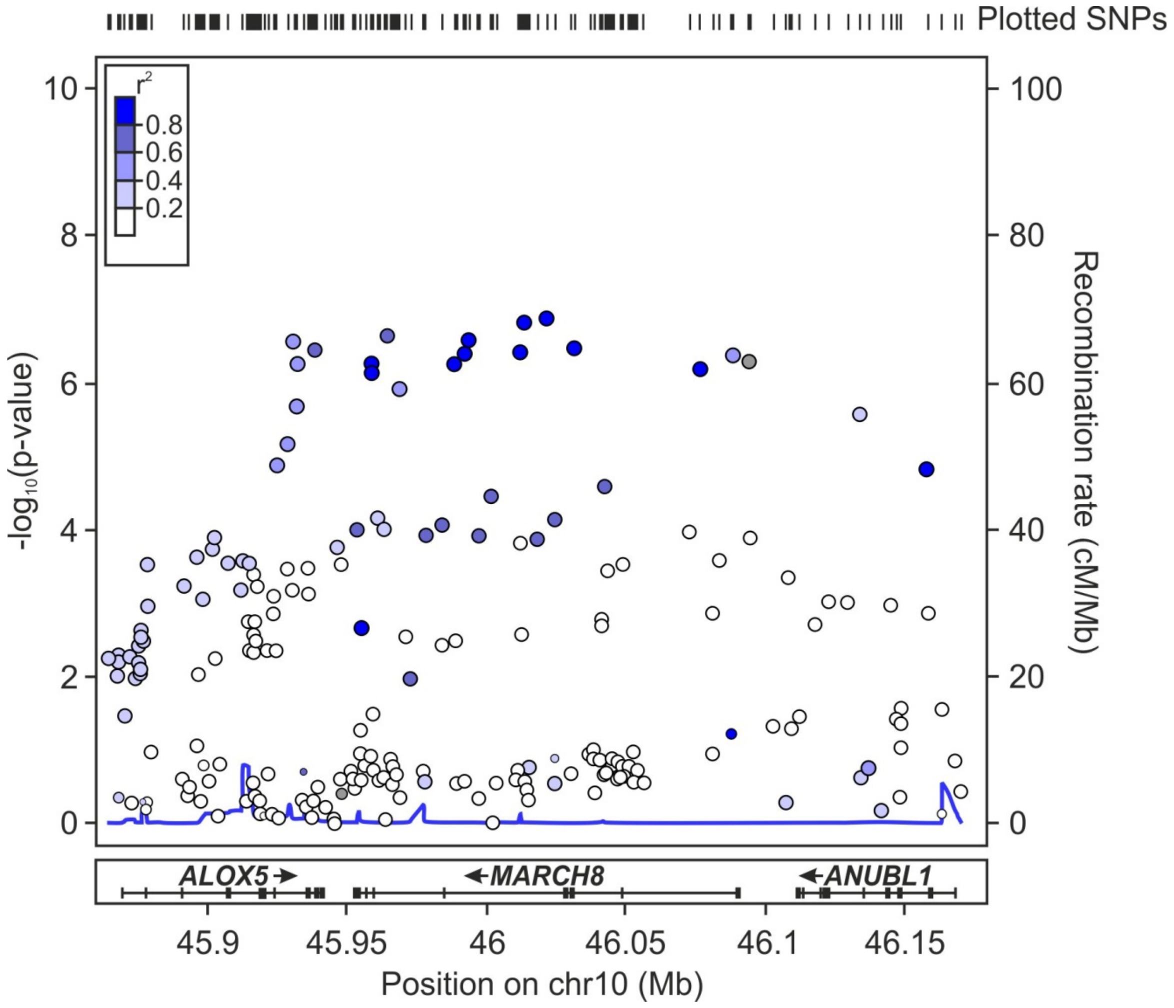
Plotted SNPs
r²
0.8
0.6
0.4
0.2
10
8
6
4
2
0
-log10(p-value)
100
80
60
40
20
0
Recombination rate (cM/Mb)
ALOX5
MARCH8
ANUBL1
45.9
45.95
46
46.05
46.1
46.15
Position on chr10 (Mb)

Figure S2

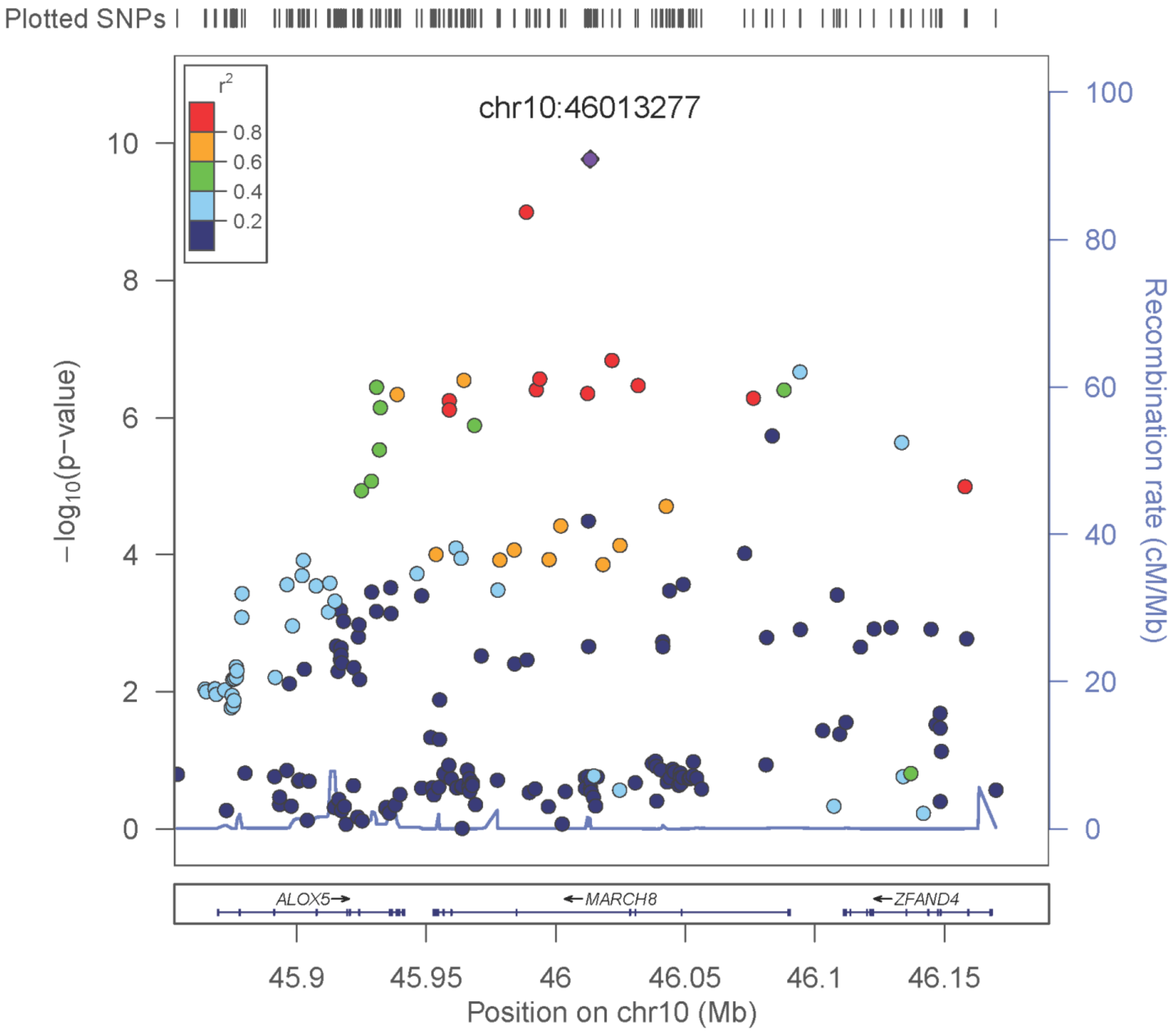

Plotted SNPs
chr10:46013277
r2
0.8
0.6
0.4
0.2
10
8
6
4
2
0
−log10(p−value)
100
80
60
40
20
0
Recombination rate (cM/Mb)
ALOX5
MARCH8
ZFAND4
45.9
45.95
46
46.05
46.1
46.15
Position on chr10 (Mb)

Figure S3

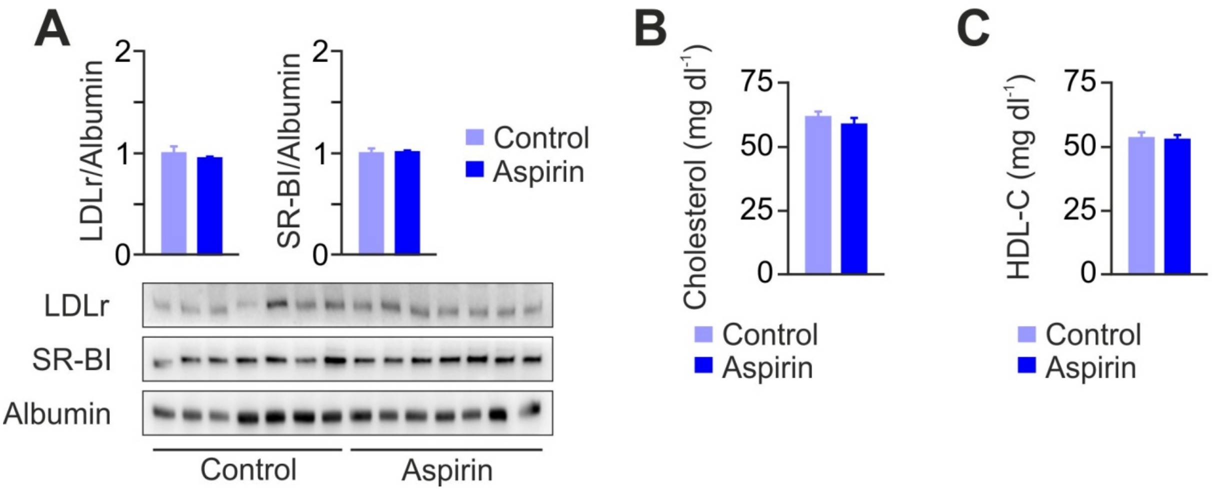

A
LDLr/Albumin
SR-BI/Albumin
Control
Aspirin
LDLr
SR-BI
Albumin
Control
Aspirin
B
Cholesterol (mg dl-1)
Control
Aspirin
C
HDL-C (mg dl-1)
Control
Aspirin

Figure S4

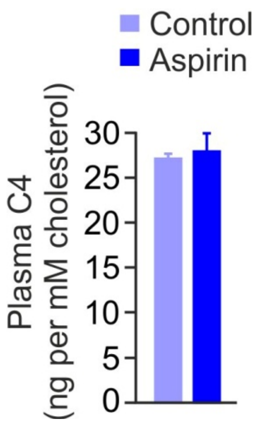

Control
Aspirin
Plasma C4
(ng per mM cholesterol)
30
25
20
15
10
5
0

Figure S5

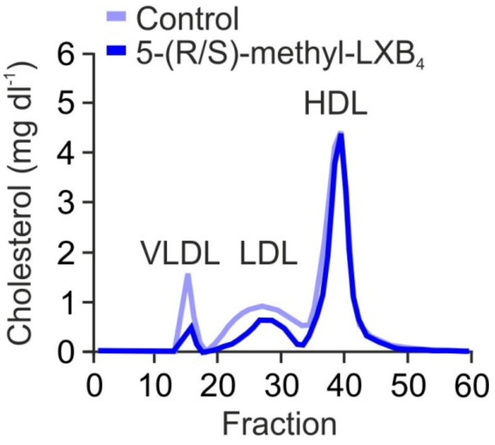
Control
5-(R/S)-methyl-LXB4
Cholesterol (mg dl-1)
6
5
4
3
2
1
0
VLDL
LDL
HDL
0
10
20
30
40
50
60
Fraction

Figure S6

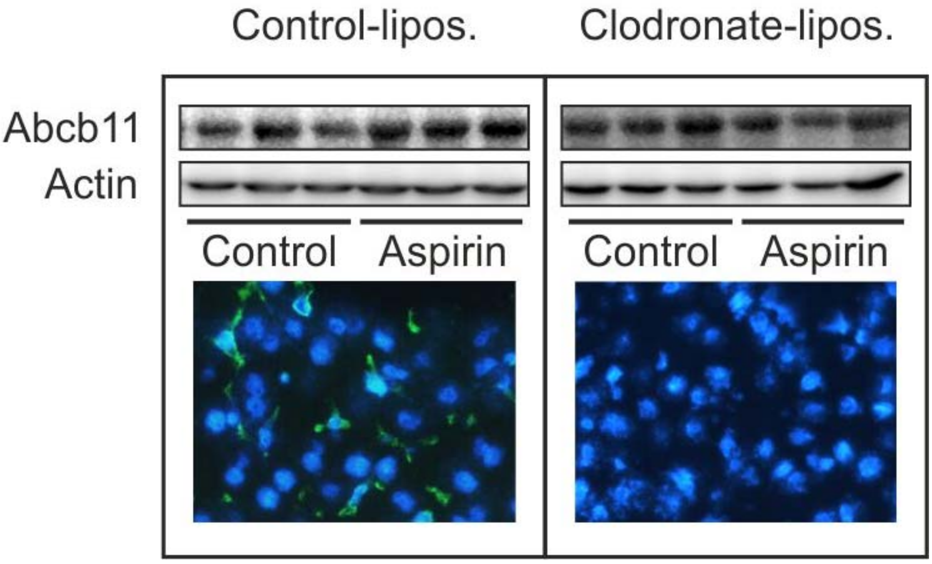

Control-lipos.
Clodronate-lipos.
Abcb11
Actin
Control
Aspirin
Control
Aspirin

Figure S7

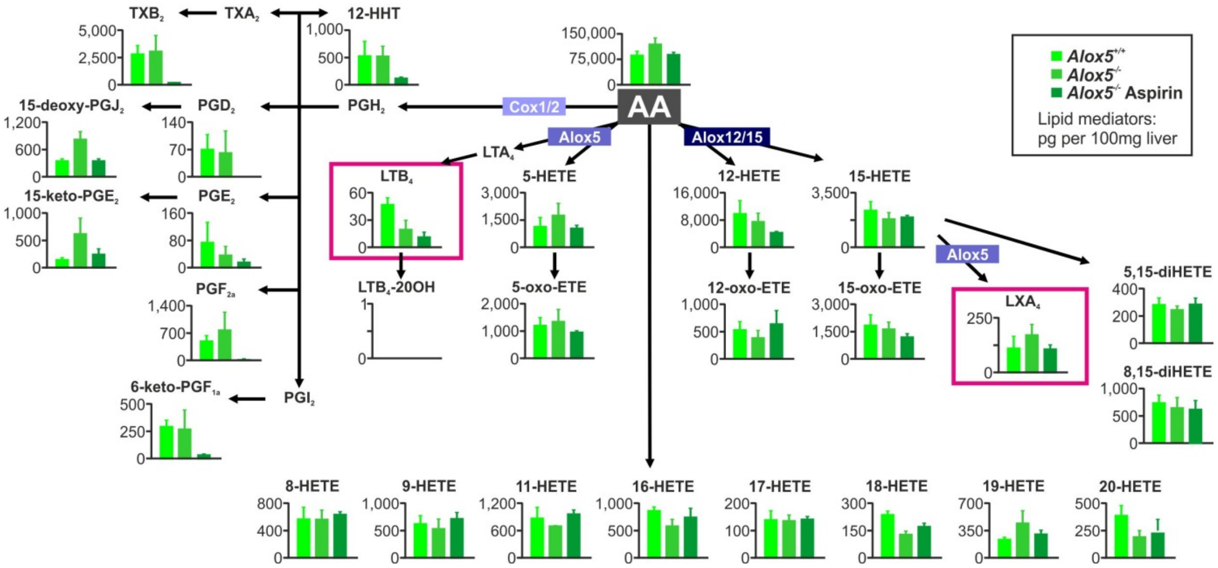

AA
Cox1/2
Alox5
Alox12/15
Alox5
TXB2
TXA2
12-HHT
PGH2
15-deoxy-PGJ2
PGD2
15-keto-PGE2
PGE2
PGF2a
6-keto-PGF1a
PGI2
LTA4
LTB4
LTB4-20OH
5-HETE
5-oxo-ETE
12-HETE
12-oxo-ETE
15-HETE
15-oxo-ETE
LXA4
5,15-diHETE
8,15-diHETE
8-HETE
9-HETE
11-HETE
16-HETE
17-HETE
18-HETE
19-HETE
20-HETE
Alox5+/+
Alox5-/-
Alox5-/- Aspirin
Lipid mediators: pg per 100mg liver
